# Observations on Those Diseases of Females Which Are Attended by Discharges; Illustrated by Copper-Plates

**Published:** 1816-05

**Authors:** 


*Observations on those Diseases of Females which are attended by Discharges; illustrated by copper-plates.* By CHARLES MANSFIELD CLARKE, &c. &c. Part I. *Mucous Discharges.* pp. 304. London.

As we mean to lay a full account of Mr. Clarke's labours before the public, we have gone rather farther back than usual for the *present volume*, in order that our analysis of the whole may be more complete.

The diseases of the sexual organs in females are certainly less known and understood by practitioners, than any other class of complaints; though they are generally distressing, and often fatal in their consequences, to those who labour under them. It is in vain for the student or practitioner to search the systems of physic or surgery for information on this head; and it requires much time, labour and expence to explore the periodical works, and detached collections and transactions of societies, for scattered observations on the diseases in question. Mr. Clarke, therefore, has conferred a great benefit on the profession at large by the course of labours he has pursued, and is pursuing, in this novel field of enquiry.

The *anatomy* of the female organs of generation is, or ought to be, well known, and therefore Mr. C. swells not his work with what should be learnt in the dissecting room. The *discharges* from these parts, though all issuing from the os externum, spring originally from various sources, and are of different kinds. 1st. From the internal surface of the uterus and Fallopian tubes. 2d. The inner membrane of the vagina. 3d. The lacunæ about the os externum. 4th. The mucous membrane of the urethra.

1. The secretions from the uterus are, α. The menstrual secretion. β. The secretion from the mucous membrane of the same. γ. The glandular secretion from the cervix of the uterus.

α. Every person knows, that the menstrual fluid is not an effusion of blood, but a *blood-coloured secretion* from the uterus. It may, however, be remarked, that when the determination of blood to the uterine vessels is increased beyond a certain point, the orifices of the vessels give way, and blood is mixed with the secreted fluid.

"If in consequence of this determination, inflammation takes place, then coagulable lymph is thrown out, and the secretion is diminished till the lamina of coagulating lymph is separated."

β. The *mucous secretion* is permanent through life, except during pregnancy. It resembles the uncoagulated white of an egg, and consists, like mucns in other parts, of albumen and soda. Its use is simply that of lubrication.

γ. The *glandular secretion* from the cervix uteri contains less water than any other mucus in the body; is semitransparent; tenacious like birdlime, and only secreted during pregnancy, especially in the first months of utero-gestation; its use is supposed to be, to prevent the ovum from escaping in its early state.

2. *Vaginal secretion.*—This mucus is naturally thinner than either of the former. It is generally in greater quantity where the rugæ of the vagina are most obliterated, as in women of lax fibre—who have borne children and who are grown old. In the latter, the rugæ have entirely disappeared.

3. *Secretion from the lacunæ of the vestibulum.*—These openings pour out a glutinous mucus which has a peculiar odour.

4. *Secretion from mucous membrane of the urethra.*—This requires no explanation.

### Chap. II. Profluvium Vaginale.

It is not of much consequence whether we denominate this discharge "γυναικεια λευκα" with Hippocrates—"Fluor muliebris" with Sydenham—"Fluor albus" with Mead—"Leucorrhœa" with Cullen, or "Sexual weakness" with Hamilton, provided we can discriminate its causes, and treat it successfully. There are objections, however, to most of the foregoing appellations, particularly those which characterize the disease as exclusively the offspring of *debility*. The author's is probably the best, and his definition is clear.

"A discharge of a fluid from the vagina, varying in its consistence, quantity, and colour; either produced by weakness of the constitution, or by a change in the structure, position, or actions of the neigbouring parts, such change being the effect of natural or morbid causes." 31.

It is important to inquire into these causes; if the discharge be the effect of mere weakness, tonics will be required; if from a tumor, its removal or support. If from inflammatory action, tonics will evidently be injurious before the topical determination is taken off. In many cases, it would be as injurious to restrain vaginal discharges, as the natural salivation in a child during dentition. *(Vide our Reviews of Dr. Parry and Dr. John Clarke.)*

The discharges from the vagina may be classed as follow: viz. 1. Transparent mucous discharge. 2. White mucous ditto. 3. Watery discharge. 4. Purulent discharge. 5. Sanguineous discharge.

The *first* is gelatinous, nearly transparent, and coagulable. The *second* is opake, perfectly white, resembling a mixture of starch and water without heat. The *third*, resembles clear water, having no colour, and very little glutinous matter. Its quantity is generally much greater than that of any of the others. The *fourth*, resembles pus secreted from the surface of an ulcer. The *fifth*, is of course, composed principally of blood. The form in which this last comes away is strictly to be attended to.

When the quantity is considerable, and from large vessels, it is fluid. When the reverse, it coagulates, assuming the figure of the parts in which it lies. When it slowly spreads itself over the surface of a tumor, it comes away in coagulated circles, which may be seen by putting the coagula in water.

The white mucous and watery discharges are pathognomonic of some uterine diseases; the sanguineous belongs to none exclusively. Of all vaginal discharges, those of mucus are the most frequent, as they are produced by several complaints of the parts, and are also the effect of mere debility in weakly constitutions. 40.

Chap. III.. This is occupied with a few general observations on sexual diseases, and on the necessity of examination *par vaginam.* The *latter* is strongly insisted on by Mr. C., though we much fear that "the general disinclination of practitioners to make an examination," will continue as long as female delicacy exists. We agree with our author, however, that the examination should be urged with firmness, whenever the case is doubtful in its nature.

"It is notorious, that many practitioners prescribe for complaints of these organs (and Mr. C. might have added many other organs) from a mere history of the symptoms given by the patient."

The fallacy of these descriptions demonstrates the futility and even injury arising from acting on such vague information.

### Chap. IV. On Sympathies.

There is very little new matter in this Chapter. We shall notice some of the sexual organ sympathies.

1. Disordered function in the *stomach,* renders the breasts flaccid, and diminishes the size of the gland itself. There is an exception to this during pregnancy, for very obvious reasons. 2. A blow on the testicles produces sickness at stomach. Functional derangement of the stomach renders the sexual passions dormant. 3. A diseased uterus produces sickness at stomach; this *last* causes *head-ache;* thus the uterus and head sympathize indirectly through the medium of the stomach. 4. The utero-gastric sympathy is the most common of all. In cancer uteri the stomach is always more or less affected with vomiting. When the uterus is ruptured, the *black vomit* comes on. 6. In amenorrhœa, and dysmenorrhœa, the utero-gastric sympathy is equally remarkable. 7. The bladder and rectum sympathize with the uterus. 8. The lower extremities, in some cases, sympathize with the uterus, before and at the time of the menses. Pain in the lower limbs has been observed as a precursor of puerperal mania. 9. The *mind* is well known to sympathize strongly with the uterus. 10. The *brain* does the same, as in furor uterinus, puerperal convulsions, and mania succeeding parturition.

### Chap. V. Diseases attended by mucous Discharges from the Vagina.

Some of these consist of the displacement of parts; as 1. Procidentia uteri. 2. Procidentia vesicæ. 3. P. vaginæ. 4. Inversio uteri.

*Procidentia uteri.*—In a healthy state of the parts, the os uteri is about an inch from the inner membrane of the vagina which forms a fold or reflection on its anterior surface. In procidentia uteri, this distance is gradually increased, according to the descent. The procidentia may be met with in all degrees till the uterus projects through the external parts, dragging with it the vagina, and forming a large tumor between the thighs of the woman. In this tumor will be contained the bladder, a portion of the rectum, and of the small intestines. Months, or even years, may elapse during this descent; for when the uterus descends to the perinæum, it there often rests, as on a cushion, for some time, the violence of the symptoms abating. The vagina, which is, of course, pushed down inverted before the womb, loses its florid colour from exposure to the air, and also its peculiar sexual irritability. The anterior part of the abdomen, from being convex, becomes flat. When the uterus and its appendages *only* have fallen out of the external parts, and before the other viscera have fallen into the inverted vagina, the tumor has a lengthened form, bearing a rude resemblance to the male organ, and giving origin to popular imposition. It may be detected by the orifice of the uterus having its *longest* diameter crosswise. As the viscera descend, the tumor becomes of a globular form. In this situation the parts contained in the tumor are liable to constriction and inflammation.

The immediate causes are, relaxation of the broad and round ligaments above; want of tone in the vagina below. Debility of the system is a predisposing or remote cause; "but the most common cause of procidentia uteri is the long-continued erect posture of the body at an early period after delivery, and in some cases, after abortion." 69. At this time the womb weighs eight or ten times more than when unimpregnated, and descends by its own gravity.

"The best practitioners direct their patients to remain in the recumbent posture upon a sofa, or on the outside of the bed; (where the advantages of horizontal posture and coolness are combined) till between the third and fourth weeks after delivery," when the uterus shall have regained its former diminution, and the ligaments and vagina their tone. A violent cough during the puerperal confinement may also produce this distressing event.

The symptoms of procidentia arise, some from dislocation of parts, others from sympathy. Pain in the back, long precedes other symptoms; also pain in the groins, extending to the labia. There is a sense of fulness in the parts, and an increased discharge of transparent mucus from the vagina. The pain in the back is next described as a *dragging* pain, with a sense of bearing down. All these are relieved by the recumbent position. To these *strangury* is frequently added.

In procidentia uteri the symptoms arising from the utero-gastric sympathy are peculiarly distressing. The appetite fails; the stomach and bowels lose their tone; flatulence and borborigmi are troublesome; the spirits flag; employment becomes irksome, and life scarcely desirable.

The discharge varies much in quantity; but with this, and the impaired digestion, great debility arises. The menstrual fluid also is increased, and menorrhagia frequently attends. *Before* the external protrusion of the tumor, the discharge is greater than *after;* because the surface of the vagina ceases to secrete, when exposed permanently to the air. Hence, in the *former* state, the patient's debility is greater than in the *latter.* Patches of healthy looking ulceration soon attack the exposed vaginal surface, but seldom go deep; the os uteri is generally assailed by one of these.

Slight degrees of procidentia uteri can only be ascertained by anatomical knowledge and manual examination.

In this disease, a tumor hangs into the vagina, or out of the os externum. But every tumor is not a prolapsed uterus. The mark of discrimination is the os uteri at the lower part of the tumor.

The *recumbent* posture relieving the symptoms of incipient proc. uteri, it is evident, that the patient should be examined both in that and in the *erect* position, if we wish to avoid deception.

*Treatment.*—If nothing be done in the way of cure, the patient generally drags on a miserable existence, for a number of years, till destroyed by accident, or some other disease. Sometimes, though rarely, debility from the discharge, or inflammation of the contents of the tumor, puts an end to her sufferings. In its early stages, it is easily relieved, and the size of the support must be in proportion to the degree of dilatation of the vagina.

If, in this situation, conception should take place, a confinement for some weeks after parturition, in the horizontal posture, will often enable the parts to regain their tone, so as to render subsequent artificial assistance unnecessary. Where pregnancy does *not* take place, we must have recourse to art.

The curative intentions are, to increase the strength of the relaxed parts, (both topically and through the constitution) and to support the tumor. *Topically*, by cold, and by astringents. Cold water ought to be applied to the female parts ; to the belly, and to the back, by means of a sponge, three or four times a day, the water being cool from the spring, or rendered colder by neutral salts. Cold water is also to be thrown into the vagina by means of a syringe. These and the horizontal position will be sufficient in slight cases. In more important cases, *astringents* are necessary. The sulphate of zinc is more useful in relaxations of the orifices of the mucous glands, than in want of tone in muscular fibres ; it is also too irritating. The same observation is applicable to nitrate of silver and sulphate of copper. The superac. plumbi is inefficacious. Solutions of alum are more powerful and less irritating than either of the foregoing. A *combination* of astringents will sometimes succeed better than any single one. Alum and zinci sulph. form a good combination in these cases. Among the vegetable astringents may be pointed out : Thea viridis ; petala rosæ rubræ ; cortex quercûs ; cortex granati ; gallæ.

In aid of topical applications, tonics must be administered internally ; especially the bitter tonics, as gentian, &c. with bark and sulphuric acid.

The bowels are to be nicely regulated ; the extremes of constipation and diarrhœa being equally injurious. Rhubarb or senna is the best purgative in these cases, the resinous being irritating ; the oily, nauseating ; and the saline debilitating. The cold-bath, where the stomach is not much weakened, previously, will be found a valuable auxiliary. The salt-water bath is preferable to the fresh. Where the former cannot be procured, the author recommends the patient to be rubbed, on coming out of the bath, with a coarse towel, which has been wrung out of a solution of salt in water, and dried at the fire.

The recumbent posture on a hard mattrass or sofa, is to be enjoined as much as possible in every case of proc. uteri, and the room of the patient kept cool.

"The diet should be nutritious, and a moderate quantity of wine may be allowed; but the stomach and intestines should never be loaded, lest the weight in the pelvis should keep up the complaint." 103.

*Pessaries.*—Those made of box wood are preferred to all others by Mr. Clarke; for the majority of cases, a circular or oval one answers sufficiently well. The circular pessary can only be used where the disease has not made much progress, and where the tone of the vagina is not much impaired.

"It will seldom be safe to introduce a circular pessary, the diameter of which exceeds two inches and a half; below this size, there can be no impropriety in using it; for it is certainly less liable to be displaced than the oval pessary. It should be large enough to keep the situation in which it is placed, else it will slip away; but it should not be so large as to distress the woman, or to injure the parts by its pressure."

Whatever may be the shape of the instrument employed as a support, it should be occasionally removed for the purpose of cleaning it; lest the secretions attached to it become acrimonious. Occasionally, also, the pessary should be changed for one of a smaller size.

The oval pessary rests with its longest diameter *across* the vagina, neither interfering with the rectum nor urinary passages. It is best adapted to those cases in which the tone of the vagina is so much diminished as to make a *large* support necessary. Its longest diameter should not exceed three inches and three-quarters. All pessaries should be introduced with care, and placed as high up in the vagina as possible.

The reduction of procidentia uteri, after its protrusion externally, must depend upon circumstances, and demands a prudent caution. Particular care should be taken to ascertain whether *inflammation* has, at any time, attacked the internal parts of the tumor; for in this case the attempt at reduction may tear open adhesions, and produce fatal, or at least, dangerous consequences. When it is thought advisable, however, to replace the tumor, the bladder and rectum are to be first emptied, and the patient so placed that the pelvis may be much higher than the shoulders.

"The practitioner is then to apply his fingers and thumb to the lower part of the tumor, where the os uteri is situated, and by a gentle pressure this is to be carried up into the centre of the tumor itself. This done, the same pressure is to be continued, and the parts are to be returned into their proper place in the pelvis.

A pessary is then to be applied, and the patient should continue in the recumbent posture for several hours.

### Chap. VIII. Procidentia Vesicæ.

This may happen at any period of life, but will be most likely to occur when the vagina is relaxed, as after child-birth. It is the posterior part of the bladder which descends; most of the patients seen by Mr. Clarke, have laboured under violent coughs. It has some symptoms in common with proc. uteri; some peculiar to itself. The sense of bearing down is not to the same extent as in proc. uteri. The size of the tumor, and the uneasiness of the part are increased when urine is contained in the bladder, and vice versâ. A mucous discharge sometimes attends. The *peculiar* symptom (not noticed by any former author) *is a pain referred to the navel, with a sense of tightness there.* This pain is greatest when the bladder is fullest, the uneasiness diminishing as the urine is evacuated, till it totally disappears. Purgatives give a *temporary,* and only a temporary relief to this symptom. The symptoms resulting from the utero-gastric sympathy, as in the former chapter, are here wanting. A distinctive mark also occurs on examination.

In procidentia uteri an opening is perceptible at the lower part of the tumor; this is not the case in procidentia vesicæ. If the *origin* of the tumor be traced, it will be felt lying between the os pubis before, and the uterus behind, and will be easily recognised as formed by a fluid. The cervix uteri is also altered in this disease. The anterior lip of the os uteri is dragged down and very much lengthened; so that in this altered state of the parts, the os uteri, instead of being felt in the centre of the pelvis, opens directly backwards, and lies in contact with the posterior part of the vagina.

"Procidentia vesicæ admits of remedy by the use of a support introduced into the vagina; and the hollow pessary, of a globular form before mentioned, is more serviceable than any other." 131.

Solutions of astringent substances should be frequently thrown into the vagina; particular care being taken to avoid straining, as every exertion of this kind must affect the displaced part, and may force away the instrument. The bowels should be kept in a relaxed state, that the fæces may pass without any great exertion. Fruit and vegetables may therefore be indulged in; and when medicines are prescribed, the mildest are to be preferred. The urinary bladder should never be allowed to be full.

### Chap. IX. Procidentia Vaginæ.

This term is meant to imply a relaxation of the posterior part of the vagina, so that this part is lower than the natural defined edge of the perinæum. From the habitual constipation of females, their false delicacy and sedentary lives, the lower part of the intestinal canal becomes so distended sometimes as to make the posterior part of the vagina approach nearer to the anterior part of the pelvis, and in this way the diameter of the vagina is much diminished. This extreme distension of the gut takes off its power of contraction, while the strength of the sphincter is increased by resistance: When, at length, by natural efforts or purgatives, the rectum is emptied, the pouch formed by it and the posterior part of the vagina continues so as to form procidentia vaginæ. It is sometimes produced by piles acting in the same manner as habitual costiveness. It produces no effect on the shape of the os uteri, and recedes a little, but never entirely, when the patient is in the horizontal position.

Very few symptoms are complained of, except some slight pain in the back, and a trifling discharge of transparent mucus.

In curing the disease the bowels are to be regulated, and the tone of the gut restored. A globular pessary gives support to the posterior part of the vagina and to the weakened rectum. Solutions of alum, in a decoction of oak bark may be thrown into the vagina several times a-day, or applied by means of a sponge. Cold water applied to the loins and to the external sexual parts, will also assist the recovery by imparting tone to the organs; but costiveness is to be most carefully guarded against. Castor oil, salts, senna, and such laxatives should be preferred, especially if there be piles, to the resinous class of purgatives.

### Chap. X. Inversio Uteri.

This complaint is the inversion of the cavity of the uterus, so that the fundus comes through the os uteri: consequently, that part which was formerly the inside of a cavity, becomes now the outside of a tumor, either in or projecting from the vagina. It is almost always the consequence of mismanagement of the placenta, and of course, in these times, a rare occurrence. Its immediate effects are hæmorrhage, faintness, and a sense of fulness in the vagina. When discovered early, it is easily reduced to its natural situation.

"This is to be effected by making pressure on the *lower part* only of the tumor, so as to make this part to be received into that above it: a continuance of the same pressing force will, in some cases, quickly reduce the tumor." 143.

If the placenta be adhering to the uterus, the latter should be reduced before any separation of the former be attempted.

This disease is occasionally met with in a chronic state, and accompanied by a mucous discharge. The symptoms resemble those of procidentia uteri. It is to be distinguished from polypus, by the os uteri encircling the latter; whereas, in inversion of the womb, the os uteri forms a part of the tumor itself. Moreover, the inverted uterus is sensible, the polypus devoid of feeling. From procidentia uteri it is distinguished by the want of an opening at the lower part; from procidentia vesicæ, by being much more resisting; by its permanence of size, and by the impossibility of finding any uterus behind it.

When it projects externally during the age of menstruation, the catamenia will be observed coming from the whole surface of the *lower part* of the tumor. Whilst the inverted uterus remains in the vagina, the discharge, excepting at the catamenial periods, will be of a mucous kind. When it protrudes, the secretion is checked by exposure to the air; but ulceration is apt to succeed, and the discharge, of course, to become purulent.

Where the uterus has been long inverted, and lies in the vagina, it will not be advisable to recommend any thing but mild astringent injections thrown into the vagina three or four times a-day; pessaries are useless, as there is no room for them.

When the inversion is in an extreme degree, the uterus hanging out of the body and dragging down with it the vagina, the discharge being great, and debility increasing, the patient's existence is either rendered miserable or cut off entirely, if the disease be left to itself. Here, if astringent and absorbent applications produce any abatement of the discharge, they should be persevered in, rather than risk an operation. But when the menstruating age is passed, and the patient's comfort is destroyed by the disease, the discharge threatening her life, it may be advisable to recommend an operation which has been successful, and from which the author knew a patient recover in her sixtieth year. How far the operation may be prudent during the period of menstruation, Mr. C. pretends not to decide. Ambrose Paré, with Mauriceau and another surgeon, cut away the womb of a woman 20 years of age, who survived; but died of pleurisy three months afterwards.

"A poor woman, 60 years of age, complained of a tumor which hung down from the external parts between her thighs, attended by a discharge of mucus and pus, so profuse in quantity as to make her exceedingly weak. Upon an examination of the tumor, it appeared to be an inverted uterus, the whole surface of which was in a state of ulceration. Above this tumor was the vagina, also inverted, having partial ulcerations upon it. The circumstances in the life of the patient obliged her to apply to the dispensary for relief, and her increasing weakness made her readily consent to an operation for the removal of the tumor, which was performed by Mr. Chevalier, surgeon to the Westminster General Dispensary. A ligature was applied round the contracted part of the tumor; that is, where the uterus ended and the vagina began. It was tightened daily, until about the eleventh or twelfth day, when the parts included in the ligature were absorbed, and the uterus fell off. During this time, the patient complained of very little pain. Adhesions had taken place between the sides of the vagina, so as to prevent the exposure of the cavity of the abdomen, and the woman recovered." 152.

### Chap. XI. On mucous discharges produced by increased determination of blood to the sexual organs.

*Hæmorrhoids.*—This disease is a dilatation of the hæmorrhoidal veins, and is more common to females than males. A discharge of mucus from the vagina is a very common accompanyment to the piles; since the internal iliac artery supplies both the hæmorrhoidal and vaginal blood-vessels; and it will be found difficult to restrain this discharge whilst the hæmorrhoidal tumors continue. The treatment of these tumors depends on the accompanying symptoms. If in a plethoric habit, and accompanied with sanguineous discharges, occasionally, it will not often be very prudent to suppress these discharges, lest some other part of the system should suffer. If the profuseness of the discharge, however, should induce debility, astringents and pressure may be safely employed. Every body knows what kind of *laxatives* (for *purgatives* are injurious) is useful in hæmorrhoids. Cold may be applied to the tumor by means of a sponge, or powdered ice may be applied between folds of linen. If the piles are external and painful, pressure may be made to return them within the sphincter, when the pain will be alleviated.

When the piles are removed, the mucous discharge often remains from the vagina. It is then to be treated by cold lotions and astringents, as before recommended.

### Chap. XII. Ascarides in the Rectum.

As mucous discharges from the urethra in men, indicative of disease in the prostate gland and testicle, are sometimes mistaken for, and treated as, a morbid state of the urethra, so mucous discharges from the vagina have been considered as originating in disease of that passage or of the uterus, when the irritation arising from ascarides in the rectum has produced all the symptoms. These worms are endowed with a very rapid motion, and have a pointed extremity with which they probably irritate the sides of the rectum, occasioning an insufferable itching, which is worse than pain to bear. This causes an increased determination of blood to the parts, and an increased secretion from the neighbouring mucous membranes. Ascarides will often travel across the perinæum from the anus to the vagina, and there occasion a similar irritation. It is not always easy to discriminate these cases, since both mucous discharge and pruritus attend many other complaints requiring opposite modes of treatment.

In children, a mucous discharge from the vagina is not very unfrequently the effect of ascarides in the rectum, or irritation in the gums.

As the rectum is the seat of ascaris, or thread-worm, the complaint may be cured by glysters, partly by mechanically washing out the gut; partly by being obnoxious to the ascarides. Solutions of bitter substances in water, as chamomile flower decoction, wormwood, or a mixture of aloes and milk, are useful in removing them. But a strong decoction of semen santonici is the most efficacious of all the injections in use; with this the rectum should be filled: But the quantity should not be so great as to produce much distention of the gut, as the latter will contract suddenly and expel the glyster, which acts with more certainty, if it remains for some time. This operation repeated for a few successive days will seldom fail to remove for a time, at least, the ascarides and the symptoms they produce.

Purgatives alone are of little service; but during the use of glysters, they are to be occasionally exhibited. Jalap, scammony, calomel are the best The *symptomatic* discharge from the vagina will disappear with its cause.

### Chap. XIII. Carcinoma Recti.

The subjects of this and the preceding chapter are introduced by Mr. C. because they are accompanied by mucous discharges from the vagina. In carcinoma recti, the capacity of the canal being diminished, the passage of the fæces through it is impeded, and painful. In consequence of this impediment in the rectum, the colon becomes greatly distended in some cases, and vast fæcal and other accumulations are occasionally found after death. The disease has often been mistaken for a stricture of the rectum, and much mischief done by attempts to dilate it by bougies. The pain attending the complaint is of the lancinating kind, and being referred to the region of the uterus has led to a supposition of the disease being there. This error, however, is not of much consequence, as the principle of treatment is the same in both cases.

In car. recti the pain is increased greatly during the passage of the fæces. In stricture the pain will be, by no means, so acute. In car. recti acute pain is also occasionally felt when no endeavour is made to expel. In stricture, the pain is only felt at this time, or for a short time afterwards. In carcinoma also, the constitution sympathises with the parts more than in stricture. In this complaint, the hæmorrhoidal veins are apt to enlarge and to bleed; the latter circumstance retards the progress of the malady. In the latter stages of carcinoma recti, and probably in the earlier, the mesenteric glands are affected according to the testimonies of writers on morbid anatomy; this accounts for the rapid progress of emaciation. The *scirrhous* state, by proper management, may be kept stationary for a considerable time. When the parts become *cancerous*, the symptoms assume a formidable aspect, and the mucous discharge is converted into a purulent one. Occasionally in the ulcerated stage, a communication is formed between the rectum and vagina; the pain becomes more acute; the stomach sympathises much, and hectic fever sometimes supervenes.

*Treatment.*—This disease admits not of *cure*, because extirpation cannot be performed; palliation is all we can afford. Stimulating applications to promote absorption of such tumors are detrimental. So are adhesive plaisters, however innocent or sedative in their natures. Common soap plaster spread upon thin linen is the best. The parts should be defended from the cold, but not kept too warm. Motion in the parts should be avoided as much as possible. Leeches are serviceable, especially, where the scirrhus is deep seated. When the skin appears on the stretch from the subjacent tumor, leeches are dangerous.

There is an intermediate stage between scirrhus and open cancer, which may be termed the *scabbing* stage, during which, the patient may live many years, with less inconvenience and pain, than in the latter part of the scirrhous stage. When the cuticle bursts, an oozing of serous matter escapes, forming, first, a film, and afterwards, a thick crust, covering even a portion of the contiguous sound surface. As long as the scab remains attached, the patient is safer than when it falls off. During the formation of this scab, the surface may be sprinkled with some light absorbent powder, as starch and oxide of zinc; the part should also be exposed to the air. All possible care is to be taken that this scab be not disturbed. These observations can only apply to carcinoma mammæ, and are a digression here; no such intermediate state is found in carcinoma of internal parts.

In treating carcinomatous tumors, every thing which can stimulate the parts, or increase the force of circulation in them, is to be carefully avoided. Where there are strength of constitution and local vascular action, blood should be taken from the region of the sacrum by the scarificators or leeches, and the operation repeated *pro re nata*. Great attention should be paid to the bowels, which are to be kept in a soluble state by expressed oils, sulphur, manna, senna, or salts. Animal food, and fermented or distilled liquors are injurious. Sexual intercourse, from the vicinity of the rectum to the vagina, should be avoided. If the bladder become irritable, the semicupium is useful. Opium should be avoided as long as possible. The mucous discharge should not be restrained by astringents, for obvious reasons; *tepid* water may be thrown up the vagina frequently, and the external parts washed with the same.

The attention of the practitioner being called to the existence of organic disease, an examination should be insisted on. Perhaps the disease may be out of reach, either by the rectum or vagina. In such cases, if astringent injections produce an augmentation of pain, it will be prudent to act as if organic disease were known to exist. Such conduct can do no harm.

To be concluded in our next.

